# Cancer-Related Cognitive Impairments (CRCIs) in Non-Central Nervous System Adult Patients: Outcome Measures and Methodology of Assessment: A Literature Review

**DOI:** 10.3390/brainsci14111119

**Published:** 2024-11-04

**Authors:** Andrea Pace, Antonio Tanzilli, Enrico Marchioni, Alessia Pellerino, Antonino Carmelo Tralongo, Paola Bini, Paolo Tralongo, Stefano Francesco Cappa

**Affiliations:** 1 IRCCS—Regina Elena National Cancer Institute, 00144 Rome, Italy; andrea.pace@ifo.it; 2 IRCCS Mondino Foundation—National Neurological Institute, 27100 Pavia, Italy; enrico.marchioni@mondino.it (E.M.); paola.bini@mondino.it (P.B.); 3 Division of Neuro-Oncology, Department of Neuroscience, University and City of Health and Science Hospital, 10126 Torino, Italy; alessia.pellerino85@gmail.com; 4 Struttura Complessa di Oncologia Medica, Ospedale Umberto I, Dipartimento di Oncologia ASP Siracusa, 96100 Siracusa, Italy; antoninocarmelo.tralongo@asp.sr.it (A.C.T.); paolo.tralongo@asp.sr.it (P.T.); 5 Department of Human and Life Sciences, University School for Advanced Studies (IUSS), 27100 Pavia, Italy; stefano.cappa@iusspavia.it; 6 IRCCS Istituto Auxologico, 20149 Milan, Italy

**Keywords:** cognitive deficit, survivorship, neurotoxicity, chemobrain

## Abstract

Background: Cancer-related cognitive impairment (CRCI) represents one of the most common and debilitating effects in patients surviving after cancer treatments. Neurocognitive deficits are important causes of disability and burden in cancer survivors. The true magnitude of CRCI is difficult to define due to significant heterogeneity of literature data. At present, there is no agreement on the gold standard for detection and grading of CRCI in clinical trials, and there is a lack of clear knowledge of its pathophysiology. Objectives: In this review, we aim to discuss some perspectives for future research to pursue in order to cover the gaps in knowledge in the CRCI field. Methods: We focused our literature research on the following relevant issues: neuroradiological correlates of CRCI; objective neuropsychological evaluation and subjective complaint assessment and their correlation with objective measures; timing of assessment; and possible treatments. Results: The correct methodology for evaluating cognitive deficits induced by anti-tumor treatments still requires a definition based on quality scientific evidence, and literature data are currently scarce. Conclusions: This review highlights the need for further research to understand the causes and consequences of cancer-related cognitive impairment using standardized and sensitive measures of cognitive functions and the long-term effects of chemotherapy on cognitive functions and to develop effective interventions.

## 1. Introduction

Neurocognitive deficits are important causes of disability and burden in non-central nervous system (non-CNS) cancer survivors. Cancer-related cognitive impairments (CRCIs) have been reported in a large percentage of cancer survivors and have become more concerning to patients and clinicians as well. The real incidence of CRCIs in cancer survival is not known due to significant heterogeneity of literature data, but more than 50% of cancer survivors complain of cognitive deterioration [1].

Indeed, the studies significantly vary regarding patient populations, intervention types, settings, and outcome measurements. At present, there is no agreement on the gold standard for detection and grading of CRCI in clinical trials, and there is a lack of clear knowledge of its pathophysiology. Several factors have been reported to contribute to the pathogenesis of CRCI, including anticancer treatments (mainly chemotherapy), age, genetic polymorphisms, and psychosocial components as well. However, other unsolved issues related to CRCIs deserving further attention include the long-term impact on cancer survivors’ quality of life (QoL) and the different perspective and education of the healthcare specialists. For these reasons, CRCIs remain a very challenging area of research as there are still several unsolved issues to be addressed in the future [2].

In this narrative review, we aim to discuss some perspectives for future research to pursue in order to cover the gaps in knowledge of the CRCI in non-CNS cancer survivors. A key point is represented by cognitive assessment in cancer patients. Moreover, as cognitive assessment can be carried out through objective or subjective scales, assessment and interpretation remains challenging. The integration of both approaches can be an important aspect to investigate if and by how CRCI is perceived differently by patients and physicians. These observations highlight the need of a core set of outcome measures for future CRCI trials. Thus, we focused our literature research on the following relevant issues: neuroradiological correlates of CRCI, objective neuropsychological evaluation and subjective complaint assessment and their correlation with objective measures, timing of assessment, and possible treatments.

### 1.1. Methods

The sources of data included the Cochrane Database of Systematic Reviews, MEDLINE, Scopus, Web of Science, PubMed, and CINAHL databases, which were searched from January 2016 to December 2022. The Cochrane database was included to obtain relevant control trials or clinical studies. MEDLINE, CINAHL, and PubMed were chosen as comprehensive databases of peer-reviewed studies from the disciplines of medicine, nursing (particularly community nursing), and allied health professions (e.g., psychology) that are most commonly associated with neurocognitive deficits in oncology. Studies using both qualitative and quantitative methods were included in the search criteria.

### 1.2. Inclusion Criteria

To be eligible for inclusion in this review, the manuscripts identified had to

- report primary data;
- include patients aged 18 years and older with cancer;
- be published in English language;
- concern patients with objective or subjective cognitive deficit

The focus of this review is on different aspects of neurocognitive status in patients with non-central nervous system cancer. For this reason, papers concerning cognitive assessment, pharmacological or non-pharmacological intervention to reduce or prevent neuropsychological deficit, and neuroimaging studies about the topic were searched. The search strategy was deliberately broad to include a wide range of tumors to ensure incorporating as many studies as possible. If there was uncertainty about whether a manuscript was relevant or not, it was decided to include it for full-text review.

### 1.3. Exclusion Criteria

The following search results were excluded from this review:

- Review papers, including systematic reviews and narrative reviews
- Single-patient case reports (case series or case studies with more than one patient were included)
- Animal studies/experimental studies
- Dissertation abstracts
- No paper available
- Book chapters/books
- Studies focusing on late effects of childhood tumors

### 1.4. Screening Process

Manuscript titles were initially screened by one author (AT, a neuropsychologist) to identify potentially relevant papers. Then, abstracts of screened studies were independently screened by AT and AP (a neurologist with decades of expertise in neuro-oncology) to identify relevant studies and assign them to the different subsessions (assessment, neuroimaging correlates, treatment, and timing). Where ambiguity regarding eligibility and assignment persisted, the full article was reviewed, and disagreements were resolved by consensus.

#### Selected Articles

The search strategy identified 562 articles in the period 2010–2022. After excluding duplicates, 134 papers met the inclusion criteria and were assigned to the subsessions according to the topic.

Forty-three articles were included in the “treatment” topic since they focused on one or more treatment strategies. A total of 39 papers evaluated assessment by using patient-reported outcome measures, and 34 approached objective assessment by means of neuropsychological tests. Fourteen studies were included among the “neuroimaging” subsession because of the comparison between CRCI and neuroradiological data. At last, 4 comprised the “timing of assessment” subgroup because of their more explicit referral to longitudinal neurocognitive assessment.

## 2. CRCI Incidence According to Different Evaluation Methods

### 2.1. Objective Neurophysiological Evaluation

Although CRCI evaluation through the means of objective neuropsychological assessment tends to yield more reliable data, drawing firm conclusions from the available literature is challenging due to the heterogeneity of studies and the multitude of variables that can potentially influence the results. Table 1 summarizes papers selected for objective neurophysiological evaluation.

Jim et al. [3] conducted a meta-analysis focused on breast cancer and standard chemotherapy, revealing a small effect size decline in verbal and visuospatial abilities. Interestingly, moderators such as age, education, time since chemotherapy, and endocrine therapy did not significantly impact the observed effects. In a high-quality meta-analysis by Linder et al. [4] that specifically examined chemotherapy effects, memory and attention impairments were found in cross-sectional studies, with progressive improvement observed in longitudinal investigations. However, moderators such as study quality and patient-related variables had no significant impact on effect sizes.

**Table 1:** Papers selected for objective neurophysiological evaluation.

Paper	Country	Population	Study Design	Sample Size
[3]	USA—Taiwan	Breast cancer	Meta-analysis	807
[5]	USA	Ovarian cancer	Cross-sectional	48
[6]	USA	Hematological-control	Prospective-longitudinal	158
[7]	USA	Multiple etiologies	Cross-sectional	657
[8]	USA—Republic of Korea	Papillary thyroid-control	Cross-sectional comparative	180
[9]	USA	Multiple etiologies	Latent class growth analysis	403
[10]	Brazil	Colorectal cancer	Observational prospective controlled	85
[11]	Italy	Multiple etiologies	Cross-sectional	136
[12]	USA	Oral-digestive cancer	Longitudinal	88

Correa’s study [5] suggested that ovarian cancer survivors may have a higher risk of developing cognitive dysfunction compared with the general population. Cognitive alterations were significantly higher among ovarian cancer survivors (28% vs. 15%). However, no differences were observed between the disease-free group and the group with active disease. Unfortunately, this study’s small sample size prevented a thorough analysis of the effects of treatments on cognitive impairment.

Syriala’s study [6] evaluated the cognitive functions of subjects over five years after allogeneic hematopoietic stem cell transplantation (HSCT) compared with matched controls. Transplantation recipients exhibited persistent deficits in motor speech fluency and dexterity, although these data did not reach sufficient statistical power. Furthermore, the study highlighted the difficulty of interpreting baseline evaluations due to potential biases caused by psychological stress related to diagnosis and imminent transplantation. Another methodological challenge in this study was the progressive reduction in the sample size during the five-year follow-up.

In Porter’s study [7], the effects of cancer on cognitive impairment were investigated in a large cohort of survivors. The study analyzed data from 9818 cancer patients from the 2006 Health and Retirement Study (HRS). The results indicated that cognitive function in the general aged population did not differ from that of cancer survivors. Moreover, the role of chemotherapy, investigated in a subsample of patients, did not reach statistical significance.

Jung et al. [8] assessed cognitive dysfunction and related risk factors in Korean women with papillary thyroid cancer compared with controls. The study found that patients with thyroid cancer performed worse in attention and working memory tasks. Additionally, patients reported more fatigue, sleep problems, and a worse self-perception of cognitive function. The study emphasized the importance of considering associated symptoms such as anxiety, depression, insomnia, and culture-specific factors such as childrearing burden when evaluating cognitive performance.

Morin’s study [9] analyzed the variation in cognitive functions, especially attention and working memory, over a six-year period in a large population with various malignancies and treatment protocols. The study found no correlation between neuropsychological scores and chemotherapy administration.

Sales et al. [10] conducted an observational prospective controlled study that described executive function deficits in subjects treated with chemotherapy for early-stage colon rectal cancer compared with the untreated group. However, the small sample size and short follow-up period limited the ability to determine whether chemotherapy-induced cognitive impairments were transient or permanent.

Muzzatti et al. [11] compared the objective and subjective perception of neuropsychological function in a group of 136 Italian long-term cancer survivors. The results indicated that verbal fluency and delayed long-term memory were the most compromised functions. Interestingly, self-perceived cognitive functioning showed a positive correlation with all investigated neuropsychological dimensions, except for total, performance, and verbal IQ estimates, highlighting that cancer survivors perceived their cognitive functions to be worse compared with individuals without cancer.

Reiger’s observational study [12] focused on veterans with various types of neoplasms. The study utilized the Montreal Cognitive Assessment (MoCA) at 6 and 18 months from diagnosis and compared the results with a control group. While the mean cognitive scores of the patients improved at 18 months compared with 6 months, they were still significantly impaired compared with controls. It is important to note that this study used only a screening measure, which may have limited its ability to detect subtle cognitive impairments. Additionally, the study did not stratify the results based on cancer heterogeneity or different treatments.

In summary, objective neuropsychological evaluation of CRCI has been investigated through various studies that have highlighted the cognitive impairments experienced by different cancer populations. These investigations have examined specific cognitive domains, and the assessment methods have included standardized neuropsychological tests and screening measures. In this regard, two important aspects hinder the possibility of obtaining shared information. Specifically, the great variety of tools available, often tailored to different aspects of the main cognitive functions, makes it difficult to compare large amounts of data. Additionally, appropriate cognitive tests should provide up-to-date norms corrected for socio-demographic and cultural effects to minimize the risk of misinterpreting patients’ cognitive performance. Unfortunately, such aspects are not always fully considered, and not all tests have normative data in all languages, thus limiting the power of conclusions. Understanding the objective neuropsychological evaluation of CRCI is crucial for identifying and managing cognitive impairments in cancer survivors.

### 2.2. Subjective Complaints’ Assessment

Several studies have explored the subjective perception of cognitive functioning in cancer survivors. The assessment by means of patient-reported outcome measures (PROms) has been increasingly utilized in oncology as important tools to know treatment- and/or disease-related consequences that are directly experienced by patients themselves, such as symptoms and health-related QoL. The use of this kind of instrument is due to their sensitivity in identifying mild and subtle cognitive sequelae. Table 2 summarizes papers selected for subjective complaints’ assessment.

In a large retrospective survey on 2537 breast cancer survivors [1], the prevalence of self-reported cognitive problems after treatment was 60%. The survey explored problems with thinking, memory, attention and trouble with concentration, short-term memory, recall, organizing daily functions, and multitasking by means of a self-assessed questionnaire. Another study evaluating self-reported perception of cognitive difficulty in a population of cancer survivors reported an incidence of about 20% without significant differences with respect to cancer-naïve controls [13].

Women often complain deficit in recalling things and people’s names they knew well before starting treatments, together with facing with well-acquired tasks, especially in case of multitasking. But, most of all, women complain a significant span reduction, with the consequence that it becomes hard to follow the plot in a movie or a book and, in general, to maintain attention focused enough to complete complex tasks [14].

These issues impact women’s life from an economic point of view since they interfere with the resumption of work and, from a psychosocial perspective, by reducing the possibility to face new challenges and increasing the suspect to have psychological disorders [15].

**Table 2:** Paper selected for Subjective complaints’ assessment.

Paper	Country	Population	Study Design	Sample Size
[1]	USA	Breast cancer	Survey	2537
[10]	Brazil	Colorectal cancer	Observational prospective controlled	85
[13]	USA	Multiple etiologies	Cross-sectional	225
[14]	Australia	Breast cancer	Qualitative-descriptive	50
[16]	USA	Breast cancer	Cross-sectional survey	357
[17]	USA	Prostate cancer	Cross-sectional	67
[18]	Canada-Australia	Colorectal cancer	Prospective-longitudinal	434
[19]	Singapore	Breast cancer	Cross-sectional	166
[20]	USA	Multiple etiologies	Cross-sectional	63
[21]	USA	Breast cancer	Cross-sectional	476
[22]	USA	Breast cancer	Cross-sectional	363

#### Factors Influencing Subjective Perception of Cognitive Impairments

Several studies analyze the interaction between self-reported cognitive deficit and factors such as anxiety, mood alterations, fatigue, or sleep disturbances that may influence subjective perception of cognitive performance. The positive correlation between distress or depression and cognitive deficit perception has been reported in different populations of cancer survivors (prostate, breast, colorectal, thyroid cancer) [16,17,18]. Fatigue and sleep disturbances have been reported to be associated with self-perception of cognitive alterations [16,19], while menopausal symptoms can contribute to CRCI without fully accounting to it [20]. Interestingly, other studies underline the impact that social or physical factors can have on self-perception of cognitive deficits. Indeed, aspects like loneliness and body mass index have been found to be positively correlated with cognitive concerns in breast cancer survivors [21,22]. Contrariwise, psychological factors such as optimism and self-affirmation can reduce the occurrence of cognitive complaints by promoting adaptive coping behaviors, and physical exercise can mitigate self-perception of cognitive deficits [22].

### 2.3. Comparison of Objective vs. Subjective Assessment

The correlation between self-reported cognitive problems and neuropsychologically evaluated cognitive problems can often be weak or absent, and self-report measures may assess different aspects of cognition than those evaluated by means of standardized neuropsychological tests [23].

Even in a longitudinal cohort study conducted in a colorectal cancer population, no significant correlation was found between perceived cognitive impairment and neurocognitive status [10], while other factors (fatigue, anxiety, depression) were associated with self-perceived deficits and worse QoL. Furthermore, contextual factors may exert an influence on objective–subjective discrepancy. Indeed, while neuropsychological assessments take place in a limited period of time, self-reported measures generally cover longer spans including, unlike formal evaluations, uncontrolled situations and unstructured environments.

According to these findings, it is important to distinguish CRCI from self-perceived symptoms and treat them as separate entities [11].

## 3. Neuroradiological Correlates of CRCI

Different multimodality magnetic resonance imaging (MRI) techniques have been investigated to evaluate the neural basis of cognitive impairment following antineoplastic treatments [24]. Table 3 summarizes papers selected for neuroradiological correlates of CRCI. Gray matter (GM), white matter (WM), cerebral blood flow, and functional connectivity changes are the main alterations that can be found in patients with cancer-induced cognitive decline. However, the MRI findings do not necessary recapitulate the scores from neurocognitive assessments and/or self-reported cognitive outcomes.

Based on the graph theory, the toxic effect of antineoplastic treatments results in an increased segregation of data analysis into the nodes and a reduction in the path length of WM across different nodal brain networks, resulting in a greater number of steps required to pass information to the neighboring regions. Lower fractional anisotropy (FA) values have been demonstrated in multiple brain regions due to a chronic inflammation of WM and brain network dysconnectivity. This means that long survivors from cancer are able to accurately complete different cognitive tasks (intact segregation), but they require more time, increased efforts, and different cognitive strategies to coordinate parallel process (impaired integration) [25]. Furthermore, a reduced nodal degree and number of hubs in dorsolateral prefrontal, orbitofrontal, and mesial temporal lobes have been reported in long survivors with breast cancer, which means impaired executive functions, memory and learning deficits, and emotional dysregulation. However, no significant associations between altered network properties and cognitive measures and/or self-reported cognitive outcome have been demonstrated thus far [26].

### 3.1. Correlations with Objective Measures

The number of chemotherapy (CT) cycles may impact GM density and WM integrity with a significant atrophy in different brain regions, leading to impaired verbal fluency and digit span performance compared with CT-naïve patients [34]. Apple et al. found that breast cancer survivors displayed a significant inward deformation in the right hippocampus, with increased global cognitive concerns and worse episodic memory performance only, while other cognitive domains were preserved in comparison with healthy controls [27]. Likewise, Kesler et al. [25] reported a significant reduction in hippocampal volumes and reduced verbal memory performance in breast cancer survivors. Interestingly, those with decreased hippocampal volumes and higher levels of tumor necrosis factor-alpha (TNFα) and lower levels of interleukin-6 (IL-6) displayed worse verbal motor functioning, suggesting a neurotoxic effect of cytokines after the end of antineoplastic treatments [25]. However, some compensatory mechanisms may be activated after a long period from the end of CT. In this regard, Conroy et al. [28] reported an increased GM density in the right frontal lobe in association with better neuropsychological performance in a cohort of breast cancer survivors after a median time of 6.4 years from the end of CT, suggesting that frontal GM may recover overtime. Similarly, patients with statistically significant WM alterations and neurocognitive deficits, which had been reported 3–4 months after the end of CT compared with baseline [24], saw an improvement of both DTI values and performance on cognitive tests after 4 years [29]. In this perspective, the reduction in FA corresponds to a CT-induced demyelination, while the longitudinal recovery of FA may indicate a neuroplastic mechanism of remyelination that may be trained with evidence-based targeted interventions to improve the axonal reorganization.

Impairments in GM and WM have been reported in patients with small-cell lung cancer (SCLC) after prophylactic cranial irradiation as well. Simò et al. [30] investigated the correlation between late cognitive effects and MRI findings in SCLC patients 2 years after prophylactic cranial irradiation. A strong correlation between lower FA score/WM damage of corpus callosum and lower scores on dementia scale was reported, while a decreased GM density was associated with impaired planning and modulation of motor pathways [30].

### 3.2. Correlations with Patient-Reported Outcome Measures

With regard to self-reported cognitive symptoms from cancer long survivors, Vardy et al. reported that breast cancer survivors who received CT and self-reported cognitive impairment had an increased frontal activation on MRI compared with those patients who received CT but reported no complaints [31]. Early and greater spatial variance of frontoparietal WM on functional MRI was correlated with executive dysfunctions in long breast cancer survivors, but not with self-perceived cognitive burden, suggesting that neurocognitive decline and self-perception of cognitive functions are two distinct phenomena [32]. In particular, self-perceived neural inefficiency may be due to psychological distress and worries regarding physical symptoms, toxic effects, changes in appearance after antineoplastic treatments, and cancer-related negative feelings, and not correlated with specific alteration of neural pathways [27,33].

Overall, different factors may influence the MRI findings, such as type and duration of antineoplastic treatments, time after the end of treatments, and the individual ability of GM/WM recovery. Last, the role of MRI in predicting the risk of development of neurocognitive impairment needs to be further investigated, as several individual and environmental factors can influence the neurocognitive recovery of long-survival patients.

## 4. Timing of Assessment

An essential aspect to consider beyond the diagnostic methods or treatment strategies in cancer survivors’ cognitive impairments is the optimal timing to assess the patients. The only data we can indirectly deduce on this question come from observational studies in which cancer survivors were tested after receiving anti-cancer treatment. A major critical point is represented by the lack of pre-treatment assessments. Cognitive concerns can manifest during or shortly after cancer treatments but also years after cancer therapy, thus suggesting an independent neurodegenerative process [35]. On the other hand, neurocognitive impairment can be found in patients with different non-CNS cancers (e.g., breast, colorectal) before the administration of systemic therapy [36,37].

Another aspect contributing to the complexity of determining the optimal timing for assessing CRCI in non-CNS cancer patients is the possibility of symptom remission.

Taken together, these factors argue in favor of the need to perform pre-chemotherapy baseline assessments with the aim to understand individual complaints and objective neurocognitive status. Subsequent evaluations, during treatment administration, are necessary to detect possible cognitive changes related to the immediate effect of chemotherapy or hormonal therapy and to possibly enable the implementation of specific neuropsychological treatments. Long time-spanning and fixed follow-ups can, instead, help in monitoring the neurocognitive status in cancer patients after the end of anti-cancer treatments.

Table 4 summarizes paper selected for timing of assessment.

Amidi et al. [38] evaluated seventy-two testicular cancer survivors starting from 2 to 7 years post-treatment. In this time window, the prevalence of cognitive impairment (verbal learning and memory, visual learning and memory, processing speed, executive functioning, and attention and working memory) was unexpectedly high, namely 62.5%. However, another experience revealed that breast cancer survivors who were more than 2 years off therapy, on average, showed lower verbal memory but no difference in executive function and verbal fluency compared with the control group [39]. Reid-Arndt et al. investigated neuropsychological functioning and quality of life during the first year after completing chemotherapy in thirty-nine breast cancer survivors. The analyses showed that no participants demonstrated subtle or more severe cognitive impairment [40].

From this evidence, it is tough to derive any recommendations: the sample size of the studies is small, the sample is predominantly based on breast cancer survivors, and the types of studies differ from each other.

The optimal timing to assess the patients remains unclear. The scant literature from which we can draw some suggestions tells us that there is probably a correct time window and that patients should not be evaluated too early (i.e., within six months a year). There is a great need to design specific studies focusing on these issues.

Uncertainty characterizes not only the incidence and severity of CRCI but also its trajectory along the disease course. While many patients report transient symptoms and a gradual reduction in deficits over time, others complain about persistent, late onset, and intermittent problems [41].

## 5. CRCI Treatment

Given that the number of etiological processes so far identified is not small, different treatments have been experimented with various outcomes.

Physical activities, cognitive rehabilitation, and cognitive-behavioral therapy have been hypothesized to have positive effects. However, other strategies such as WEB-based or computer-based training programs and acupuncture have been taken into consideration, showing promising results. In this setting, the rule of pharmacological intervention needs to be better investigated.

Table 5 summarizes papers selected for CRCI treatment.

### 5.1. Physical Activity

Physical activity includes several types of interventions, which are different in duration and intensity and are time-consuming, and the outcomes are not all the same. According to Ehlers et al., moderate-to-vigorous physical activity seems to be associated with a better performance in cognitive tasks, while light-intensity physical activity performance is worse than sedentary time [42]. Another recent study has confirmed moderate-to-vigorous physical activity as a useful intervention to improve depression and its related cognitive impairment [43].

Focusing on lifestyle interventions, different expressions of physical activities have been investigated, reporting heterogeneous outcomes.

A randomized crossover trial analyzed the effect of moderate-intensity treadmill walking or 30 min of seated rest in twenty-seven breast cancer survivors. The results showed an improvement in reaction time on processing speed and better accuracy on spatial working memory in pre- and post-rest seated sessions, respectively [44].

A randomized controlled trial (RCT) in postmenopausal breast cancer survivors with self-reported cognitive dysfunction following chemotherapy treatment investigated aerobic exercise role and its impact on the quality of life. The intervention group performed better just in 1 objective neuropsychological test, and no differences were reported between the two arms in PROMs [45].

Neither moderate walking levels, compared with usual care, in breast cancer patients treated with chemotherapy seems to produce modest benefits on objective neuropsychological measures, although the positive effect is represented by better psychosocial functioning, and exercise can be useful in protecting against a decline in subjective cognitive impairments [46].

Nevertheless, the impact of physical exercise on self-reported cognitive complaints is not confirmed by other reports [47].

#### Other Types of Physical Exercise

Three different experiences were found in this setting:

The effect of YOGA (Iyenger-inspired program twice a week for 12 weeks) was evaluated in a case series study, including four early-stage breast cancer patients, to assess its impact on cognition and life quality. The cognition measures were collected with the Perceived Cognition Questionnaire (PCQ) and CogState. The patients analyzed showed an improvement in neuropsychological performances [48].

A significant effect of Qigong (a mindfulness-based form of exercise) compared with survivorship support was evidenced by PROMs, and a partial improvement was found in objective tests (Trail Making A and B test) [49].

Twenty-three women with a history of cancer participated in 60 min of Tai Chi. Promising results on neuropsychological benefits (immediate and delayed memory, verbal fluency, attention, executive functioning, stress levels, and vigor) also derive from practicing Tai Chi [50].

The impact of these results is very modest, and the strength of the conclusions is minimal. All these studies have a small sample size with limited power to capture differences among the groups analyzed, and the population is hyperselected (most of all highly educated breast cancer patients). Moreover, it is unclear if the test battery used is sufficiently sensitive to detect improvements, and the intervention may not have been long enough to catch the impact of physical activity on cognition.

### 5.2. Cognitive Rehabilitation

Several studies have explored the role of cognitive rehabilitation in group sessions. It was achieved in different manners. Ercoli et al. have demonstrated the feasibility of a rehabilitation intervention program for breast cancer survivors with cognitive impairment, observing a significant improvement in neurocognitive tests and reducing total and memory-specific cognitive complaints [51]. Feasibility was investigated by two other studies as well, showing an improvement effect of group cognitive rehabilitation in the outcomes analyzed [52,53]. Another similar and more recent study in which a manualized cognitive rehabilitation program was used showed an improvement trend across time on most of the neuropsychological measures [54].

The efficacy of group cognitive rehabilitation was shown in different prospective studies.

A 4-week group cognitive rehabilitation treatment focusing on cognition, problem solving, memory, and attention was evaluated in thirty-two cancer patients. The intervention was associated with improved objective and subjective cognition, psychosocial distress, social functioning, and perceived understanding of cognition [55].

These results on objective and subjective measures have been confirmed by an RCT analyzing a 7-week group cognitive rehabilitation intervention in twenty-eight cancer survivors [56].

A further confirmation of group cognitive rehabilitation efficacy derives from an RCT of twenty-nine breast cancer survivors. The results showed immediate and sustained improvements in self-reported cognitive complaints and memory functioning on neurocognitive testing [57].

Sample sizes of these studies are small, limiting the statistical power to detect the differences among the groups considered. Moreover, the first study reported was not randomized. In the last trial, there was some imbalance in the baseline characteristics of the two groups.

### 5.3. WEB or PC Program Rehabilitation

WEB-based or computer-based programs developed to help this setting of patients in their mental issues have been tested more and more in the recent past. These programs incorporate psychoeducation, skill training, strategy training, and functional activity training to apply the strategies in everyday life.

Kesler et al. conducted an RCT, testing the feasibility and effectiveness of an online executive function program in long-term breast cancer survivors and improving cognitive flexibility, verbal fluency, and processing speed [58].

In another RCT, a WEB-based cognitive rehabilitation intervention produced a significant reduction in prospective memory failures and a positive trend on perceived cognitive impairments and executive function [59].

Meneses et al. enrolled sixty breast cancer survivors in a randomized controlled pilot, testing a WEB-based program (SOP training), finding an improvement in the intervention group in the episodic memory, executive function, and four subtests of the useful field-of-view (UFOV) test [60].

A 15-week home-based intervention based on a computerized neurocognitive learning program was proposed to 122 cancer survivors. An improvement in perceived cognitive function, anxiety, fatigue, depression, stress, and quality of life was noted, but no difference in the objective neuropsychological tests was found [61].

An RCT WEB-based cognitive training program on 46 breast cancer patients (23 early treated for 6 months and 23 with delayed treatment for 3 months) confirmed the positive impact of treatment on objective and subjective measures [69].

In a mixed method study on 25 breast cancer survivors, computer-assisted cognitive training and computer-assisted cognitive training with music were tested. The addition of music determined significant improvements in self-perceived cognitive ability [70].

These trials’ results are weak for several reasons: most of them have a small sample size; often, the experimental group performed the programs at home, and an uncontrolled environment and performance on tasks were susceptible to unknown influences. Finally, the validation of the training programs analyzed remains unclear.

### 5.4. Cognitive-Behavioral Therapy

Data from one RCT showed a positive effect of CBT in reducing concentration problems and self-reported cognitive disability, even though no difference in neuropsychological test performance was recorded. The CBT focused on six perpetuating factors for post-cancer fatigue, measured with specific questionnaires [62].

Two RCTs tested as intervention a videoconference-delivered CBT, in contrast to computer-based cognitive rehabilitation, characterized by repetitive practice of cognitive exercises that may not be generalizable to daily life. The first used the Memory and Attention Adaptation Training (MAAT) program, confirming significant gains in self-reported cognitive impairments and cognitive processing speed compared with the control treatment. The second one used telemedicine sessions (in comparison with a session group in a community center and a waitlist control group). This strategy improved the cognitive function, fatigue, sleep disturbance, and mental-health-related and breast-cancer-related quality of life [63,64].

To improve cancer-related fatigue, thirteen breast cancer survivors were enrolled in an RCT and randomized to four weekly 50-min sessions of CBT (optimizing exercise routines, improving nutrition, and implementing evidence-based cognitive-behavioral technique) or a control group. Participants in the intervention group experienced a 66% reduction in fatigue [64].

### 5.5. Other Interventions

A recent Chinese experience experimented the manual acupuncture provided by a single acupuncturist trained in traditional Chinese medicine in fifteen women with gynecological cancer, stage I–III, ready for adjuvant chemotherapy. Compared with the cancer control group, the results showed that acupuncture improved neurocognitive performance over the chemotherapy period [65]. Acupuncture seems to have a positive effect even if associated with cognitive-behavioral therapy [66].

EEG biofeedback (neurofeedback) is a real-time display of the brain’s electrical activity fed back as visual or auditory information enabling the user to modify brainwave activity. Limited evidence related to this strategy still exists. Alvarez et al. demonstrated the feasibility and effectiveness of neurofeedback in reducing the cognitive impairment of the sample analyzed [67].

### 5.6. Psychostimulants

Different agents were tested: donepezil, methylphenidate, modafinil, armodafinil.

Lawrence et al. analyzed sixty-two patients in an RCT to test 5 mg of donepezil/day vs. placebo for six weeks and, if well tolerated, 10 mg/day for 18 weeks. At the end of the study, the experimental group performed better just on two parameters of memory. No significant side-effect-related issues were reported [68].

A recent meta-analysis was conducted to better understand if psychostimulants are useful in cancer survivors’ cognitive impairments, assuming the primary outcome’s cognitive functions change. However, the authors concluded that the important differences in methodologies among the studies included made it inappropriate to conduct a meta-analysis. The evidence is too limited to estimate these agents’ real usefulness on cognitive function in this setting of population [71].

Regardless of the specific drugs and physical activity tested, and despite the fact that their involvement in neurotransmitter pathways has been hypothesized, the exact mechanisms of action are not completely clear. With regard to cognitive treatments, the mechanisms rely on the principles of neural plasticity, and two mechanisms of functional reorganization have been found: (1) recruitment of residual dominant hemisphere structures and (2) recruitment of nondominant hemisphere areas [72].

### 5.7. Conclusions

Cognitive deficits are quite common in people undergoing, or who have undergone, oncological treatment (i.e., chemotherapy and immunotherapy) and can have a deleterious impact on their quality of life. Multiple factors influence cognitive outcomes in cancer patients and interact in a complex manner. Studies on this topic differ in methodologies, sample sizes, tools used, and interventions proposed, thus reducing the possibility to make comparisons and to draw firm conclusions [4].

Methodological and study population differences, together with treatment and patient-specific variables, can account, at least in part, for the conflicting results.

Many unanswered questions, for example, cognitive reserve and socioeconomic status, remain, and their influence on cognition and recovery have not been well studied.

Despite the progress in the field, made utilizing neuropsychological tests, concerns have been raised about the sensitivity of these tools to detect the subtle neuropsychological deficits frequently affecting cancer patients and survivors.

Moreover, high-quality evidence on how to help cancer survivors dealing with cognitive complaints is scarce, and no drugs have been approved to reduce or prevent them since no evidence in support of their use has emerged [73]. On the other hand, studies on cognitive improvement due to physical exercise show limited results [44], and evidence from cognitive rehabilitation reports [51,54,56] is promising but still limited. Because of the association of neurocognitive symptoms with psychological complaints (anxiety, depression) and with fatigue, and sleep disorder is frequent, it is important to perform complete and multidisciplinary assessment involving different specialists in the team. Particular attention should be paid on how these factors interplay especially with self-reported cognitive complaints. A recent cross-sectional analysis evidenced how fatigue and anxiety are associated with an increased risk of perceived attention impairment, while anxiety is one of the most important factors in perceived memory impairment [74]. In this perspective, a comprehensive evaluation of this complex interaction should comprise patients’ self-administered measures but also objective assessment measures and caregiver contribution [75].

Early detection of cognitive deficit can be particularly important, especially in elderly patients in order to refer them to an onco-geriatry or neurology services to help manage cognitive impairment before and during treatment.

The correct methodology for evaluating cognitive deficits induced by anti-tumor treatments is an aspect that still requires a definition based on quality scientific evidence, and literature data are currently scarce. Evaluation with longitudinal neuropsychological tests is considered the most scientifically valid objective method, but measurements based on patient perception (PROM) that use different self-report assessment tools of specific symptoms as functional assessment of cancer therapy—cognitive (FACT-Cog), Short Form Health Survey 36 (SF-36), or their impact on quality of life (EORTC QLQ-C 30)—appear to have greater sensitivity in identifying the presence of mild/moderate cognitive deficits and the effectiveness of treatments.

This review highlights the need for further research to understand the causes and consequences of cancer-related cognitive impairment using standardized and sensitive measures of cognitive functions and the long-term effects of chemotherapy on cognitive functions, and to develop effective interventions. Moreover, it highlights the fact that management of cancer-related cognitive impairment should become part of the clinical practice for patients with other types of neurological disease.

## 6. Several Issues Need to Be Addressed in Future Studies

There is no general agreement on the diagnostic assessment of CRCI. Primarily, literature reports are heterogeneous since they did not follow common criteria for the disease course evaluation and the stratification for sex, cancer, and treatments. Moreover, methodological limitations, such as the use of qualitative observational, cross-sectional designs and the large variability of tools, do not allow clear conclusions about the real incidence and severity of CRCI. The poor prognosis that characterizes most etiologies hinders the conduction of longitudinal studies on large samples of patients. Another key matter is that most of the studies concern breast cancer patients, while other cancer patients are under-represented. At last, baseline assessments (especially in elderly subjects) is almost always absent, and other systemic or CNS diseases such as metastases or radio-induced brain damages are rarely considered.

This prevents us to evaluate the impact of possible previous cognitive impairment.

## Figures and Tables

**Table 3:** Paper selected for Neuroradiological correlates of CRCI.

Paper	Country	Population	Study Design	Sample Size
[24]	Belgium	Breast cancer	Longitudinal	34
[25]	USA	Breast cancer	Cross-sectional	77
[26]	USA	Breast cancer	Cross-sectional	61
[27]	USA	Breast cancer	Cross-sectional	34
[28]	USA	Breast cancer	Cross-sectional	47
[29]	Belgium	Breast cancer	Cross-sectional, longitudinal	54
[30]	Spain	Lung cancer	Cross-sectional	22
[31]	Australia—Canada	Breast cancer	Cross-sectional	126
[32]	Republic of Korea, USA	Breast cancer	Prospective, longitudinal	92
[33]	USA	Breast cancer	Cross-sectional	50

**Table 4:** Paper selected for timing of assessment.

Paper	Country	Population	Study Design	Sample Size
[38]	Denmark, USA	Testicular cancer	Cross-sectional	72
[39]	USA	Breast cancer	Observational-retrospective	62
[40]	USA	Breast cancer	Longitudinal	46

**Table 5:** Paper selected for CRCI Treatment.

Paper	Country	Population	Study Design	Sample Size
[42]	USA	Breast cancer	Cross-sectional	271
[43]	USA	Breast cancer	Cross-sectional	317
[44]	USA	Breast cancer	Repeated, crossover	27
[45]	Canada	Breast cancer	RCT	19
[46]	UK	Breast cancer	RCT	50
[47]	USA	Breast cancer	RCT	87
[48]	USA	Breast cancer	Case series	4
[49]	USA	Breast cancer	Randomized single-blind, three arm	50
[50]	USA	Multiple etiologies	Longitudinal	23
[51]	USA	Breast cancer	Pilot study	27
[52]	Australia	Breast cancer	Observational	27
[53]	USA	Gynecologic cancer	Pilot study	12
[54]	Canada	Breast cancer	Pilot study	12
[55]	Australia	Multiple etiologies	Nonrandomized	46
[56]	USA	Multiple etiologies	RCT	28
[57]	USA	Breast cancer	RCT	48
[58]	USA	Breast cancer	RCT	41
[59]	Australia	Breast cancer, leukemia	RCT	76
[60]	USA	Breast cancer	RCT pilot	60
[61]	Australia	Multiple etiologies	RCT	243
[62]	The Netherlands	Multiple etiologies	RCT	98
[63]	USA	Breast cancer	RCT	47
[64]	USA	Breast cancer	RCT	102
[65]	USA	Breast cancer	RCT	13
[66]	China	Gynecologic cancer	Prospective cohort study	30
[67]	USA	Breast cancer	Prospective	23
[68]	USA	Breast cancer	RCT	47

## Data Availability

Data will be available upon reasonable request.

## References

[1] Buchanan N.D., Dasari S., Rodriguez J.L., Lee Smith J., Hodgson M.E., Weinberg C.R., Sandler D.P. (2015). Post-treatment Neurocognition and Psychosocial Care Among Breast Cancer Survivors. Am. J. Prev. Med..

[2] Oliva G., Giustiniani A., Danesin L., Burgio F., Arcara G., Conte P. (2024). Cognitive impairment following breast cancer treatments: An umbrella review. Oncologist.

[3] Jim H.S., Phillips K.M., Chait S., Faul L.A., Popa M.A., Lee Y.H., Hussin M.G., Jacobsen P.B., Small B.J. (2012). Meta-analysis of cognitive functioning in breast cancer survivors previously treated with standard-dose chemotherapy. J. Clin. Oncol..

[4] Lindner O.C., Phillips B., McCabe M.G., Mayes A., Wearden A., Varese F., Talmi D. (2014). A meta-analysis of cognitive impairment following adult cancer chemotherapy. Neuropsychology.

[5] Correa D.D., Zhou Q., Thaler H.T., Maziarz M., Hurley K., Hensley M.L. (2010). Cognitive functions in long-term survivors of ovarian cancer. Gynecol. Oncol..

[6] Syrjala K.L., Artherholt S.B., Kurland B.F., Langer S.L., Roth-Roemer S., Elrod J.B., Dikmen S. (2011). Prospective neurocognitive function over 5 years after allogeneic hematopoietic cell transplantation for cancer survivors compared with matched controls at 5 years. J. Clin. Oncol..

[7] Porter K.E. (2013). “Chemo brain”—Is cancer survivorship related to later-life cognition? Findings from the health and retirement study. J. Aging Health.

[8] Jung M.S., Visovatti M. (2017). Post-treatment cognitive dysfunction in women treated with thyroidectomy for papillary thyroid carcinoma. Support. Care Cancer.

[9] Morin R.T., Midlarsky E. (2018). Treatment With Chemotherapy and Cognitive Functioning in Older Adult Cancer Survivors. Arch. Phys. Med. Rehabil..

[10] Sales M.V.C., Suemoto C.K., Apolinario D., Serrao V., Andrade C.S., Conceição D.M., Amaro E., de Melo B.A.R., Riechelmann R.P. (2019). Effects of Adjuvant Chemotherapy on Cognitive Function of Patients With Early-stage Colorectal Cancer. Clin. Color. Cancer.

[11] Muzzatti B., Giovannini L., Flaiban C., Cattaruzza N., Annunziata M.A. (2019). Cognitive functioning in long-term cancer survivorship: A survey utilizing both standardized neuropsychological and self-report measures. Appl. Neuropsychol. Adult.

[12] Regier N.G., Naik A.D., Mulligan E.A., Nasreddine Z.S., Driver J.A., Sada Y.H., Moye J. (2019). Cancer-related cognitive impairment and associated factors in a sample of older male oral-digestive cancer survivors. Psychooncology.

[13] Braun I.M., Rao S.R., Pirl W.F. (2012). Comparison of self-reported cognitive difficulties in a national sample of long-term cancer survivors and cancer-naive controls. Psychosomatics.

[14] Bolton G., Isaacs A. (2018). Women’s experiences of cancer-related cognitive impairment, its impact on daily life and care received for it following treatment for breast cancer. Psychol. Health Med..

[15] Yamamoto S., Masutani E., Arao H. (2020). Self-reported cognitive decline in Japanese patients with breast cancer treated with endocrine therapy. Breast Cancer.

[16] Seliktar N., Polek C., Brooks A., Hardie T. (2015). Cognition in breast cancer survivors: Hormones versus depression. Psychooncology.

[17] Eisenberg S.A., Kurita K., Taylor-Ford M., Agus D.B., Gross M.E., Meyerowitz B.E. (2015). Intolerance of uncertainty, cognitive complaints, and cancer-related distress in prostate cancer survivors. Psychooncology.

[18] Dhillon H.M., Tannock I.F., Pond G.R., Renton C., Rourke S.B., Vardy J.L. (2018). Perceived cognitive impairment in people with colorectal cancer who do and do not receive chemotherapy. J. Cancer Surviv..

[19] Cheung Y.T., Shwe M., Chui W.K., Chay W.Y., Ang S.F., Dent R.A., Yap Y.S., Lo S.K., Ng R.C., Chan A. (2012). Effects of chemotherapy and psychosocial distress on perceived cognitive disturbances in Asian breast cancer patients. Ann. Pharmacother..

[20] Vega J.N., Dumas J., Newhouse P.A. (2018). Self-reported chemotherapy-related cognitive impairment compared with cognitive complaints following menopause. Psychooncology.

[21] Jaremka L.M., Peng J., Bornstein R., Alfano C.M., Andridge R.R., Povoski S.P., Lipari A.M., Agnese D.M., Farrar W.B., Yee L.D. (2014). Cognitive problems among breast cancer survivors: Loneliness enhances risk. Psychooncology.

[22] Myers J.S., Wick J.A., Klemp J. (2015). Potential factors associated with perceived cognitive impairment in breast cancer survivors. Support. Care Cancer.

[23] O’Farrell E., Smith A., Collins B. (2017). Objective-subjective disparity in cancer-related cognitive impairment: Does the use of change measures help reconcile the difference?. Psychooncology.

[24] Deprez S., Amant F., Smeets A., Peeters R., Leemans A., Van Hecke W., Verhoeven J.S., Christiaens M.R., Vandenberghe J., Vandenbulcke M. (2012). Longitudinal assessment of chemotherapy-induced structural changes in cerebral white matter and its correlation with impaired cognitive functioning. J. Clin. Oncol..

[25] Kesler S., Janelsins M., Koovakkattu D., Palesh O., Mustian K., Morrow G., Dhabhar F.S. (2013). Reduced hippocampal volume and verbal memory performance associated with interleukin-6 and tumor necrosis factor-alpha levels in chemotherapy-treated breast cancer survivors. Brain Behav. Immun..

[26] Bruno J., Hosseini S.M., Kesler S. (2012). Altered resting state functional brain network topology in chemotherapy-treated breast cancer survivors. Neurobiol. Dis..

[27] Apple A.C., Ryals A.J., Alpert K.I., Wagner L.I., Shih P.-A., Dokucu M., Cella D., Penedo F.J., Voss J.L., Wang L. (2017). Subtle hippocampal deformities in breast cancer survivors with reduced episodic memory and self-reported cognitive concerns. Neuroimage Clin..

[28] Conroy S.K., McDonald B.C., Smith D.J., Moser L.R., West J.D., Kamendulis L.M., Klaunig J.E., Champion V.L., Unverzagt F.W., Saykin A.J. (2013). Alterations in brain structure and function in breast cancer survivors: Effect of post-chemotherapy interval and relation to oxidative DNA damage. Breast Cancer Res. Treat..

[29] Billiet T., Emsell L., Vandenbulcke M., Peeters R., Christiaens D., Leemans A., Van Hecke W., Smeets A., Amant F., Sunaert S. (2018). Recovery from chemotherapy-induced white matter changes in young breast cancer survivors?. Brain Imaging Behav..

[30] Simó M., Vaquero L., Ripollés P., Jové J., Fuentes R., Cardenal F., Rodríguez-Fornells A., Bruna J. (2016). Brain damage following prophylactic cranial irradiation in lung cancer survivors. Brain Imaging Behav..

[31] Vardy J.L., Stouten-Kemperman M.M., Pond G., Booth C.M., Rourke S.B., Dhillon H.M., Dodd A., Crawley A., Tannock I.F. (2019). A mechanistic cohort study evaluating cognitive impairment in women treated for breast cancer. Brain Imaging Behav..

[32] Jung M.S., Zhang M., Askren M.K., Berman M.G., Peltier S., Hayes D.F., Therrien B., Reuter-Lorenz P.A., Cimprich B. (2017). Cognitive dysfunction and symptom burden in women treated for breast cancer: A prospective behavioral and fMRI analysis. Brain Imaging Behav..

[33] Berman M.G., Askren M.K., Jung M., Therrien B., Peltier S., Noll D.C., Zhang M., Ossher L., Hayes D.F., Reuter-Lorenz P.A. (2014). Pretreatment worry and neurocognitive responses in women with breast cancer. Health Psychol..

[34] Li X., Chen H., Lv Y., Chao H.H., Gong L., Li C.-S.R., Cheng H. (2018). Diminished gray matter density mediates chemotherapy dosage-related cognitive impairment in breast cancer patients. Sci. Rep..

[35] Parsons M.W., Dietrich J. (2019). Assessment and management of cognitive changes in patients with cancer. Cancer.

[36] Wefel J.S., Kayl A.E., Meyers C.A. (2004). Neuropsychological dysfunction associated with cancer and cancer therapies: A conceptual review of an emerging target. Br. J. Cancer.

[37] Ahles T.A., Saykin A.J., McDonald B.C., Furstenberg C.T., Cole B.F., Hanscom B.S., Mulrooney T.J., Schwartz G.N., Kaufman P.A. (2008). Cognitive function in breast cancer patients prior to adjuvant treatment. Breast Cancer Res. Treat..

[38] Amidi A., Wu L.M., Pedersen A.D., Mehlsen M., Pedersen C.G., Rossen P., Agerbæk M., Zachariae R. (2015). Cognitive impairment in testicular cancer survivors 2 to 7 years after treatment. Support Care Cancer.

[39] Kesler S.R., Blayney D.W. (2016). Neurotoxic Effects of Anthracycline- vs Nonanthracycline-Based Chemotherapy on Cognition in Breast Cancer Survivors. JAMA Oncol..

[40] Reid-Arndt S.A., Hsieh C., Perry M.C. (2010). Neuropsychological functioning and quality of life during the first year after completing chemotherapy for breast cancer. Psychooncology.

[41] Ng T., Dorajoo S.R., Cheung Y.T., Lam Y.C., Yeo H.L., Shwe M., Gan Y.X., Foo K.M., Loh W.K., Koo S.L. (2018). Distinct and heterogeneous trajectories of self-perceived cognitive impairment among Asian breast cancer survivors. Psychooncology.

[42] Ehlers D.K., Fanning J., Salerno E.A., Aguiñaga S., Cosman J., Severson J., Kramer A.F., McAuley E. (2018). Replacing sedentary time with physical activity or sleep: Effects on cancer-related cognitive impairment in breast cancer survivors. BMC Cancer.

[43] Bedillion M.F., Ansell E.B., Thomas G.A. (2019). Cancer treatment effects on cognition and depression: The moderating role of physical activity. Breast.

[44] Salerno E.A., Rowland K., Kramer A.F., McAuley E. (2019). Acute aerobic exercise effects on cognitive function in breast cancer survivors: A randomized crossover trial. BMC Cancer.

[45] Campbell K.L., Kam J.W.Y., Neil-Sztramko S.E., Ambrose T.L., Handy T.C., Lim H.J., Hayden S., Hsu L., Kirkham A.A., Gotay C.C. (2018). Effect of aerobic exercise on cancer-associated cognitive impairment: A proof-of-concept RCT. Psychooncology.

[46] Gokal K., Munir F., Ahmed S., Kancherla K., Wallis D. (2018). Does walking protect against decline in cognitive functioning among breast cancer patients undergoing chemotherapy? Results from a small randomized controlled trial. PLoS ONE.

[47] Hartman S.J., Nelson S.H., Myers E., Natarajan L., Sears D.D., Palmer B.W., Weiner L.S., Parker B.A., Patterson R.E. (2018). Randomized controlled trial of increasing physical activity on objectively measured and self-reported cognitive functioning among breast cancer survivors: The memory & motion study. Cancer.

[48] Galantino M.L., Greene L., Daniels L., Dooley B., Muscatello L., O’Donnell L. (2012). Longitudinal impact of yoga on chemotherapy-related cognitive impairment and quality of life in women with early stage breast cancer: A case series. Explore.

[49] Myers J.S., Mitchell M., Krigel S., Steinhoff A., Boyce-White A., Van Goethem K., Valla M., Dai J., He J., Liu W. (2019). Qigong intervention for breast cancer survivors with complaints of decreased cognitive function. Support Care Cancer.

[50] Reid-Arndt S.A., Matsuda S., Cox C.R. (2012). Tai Chi effects on neuropsychological, emotional, and physical functioning following cancer treatment: A pilot study. Complement. Ther. Clin. Pract..

[51] Ercoli L.M., Castellon S.A., Hunter A.M., Kwan L., Kahn-Mills B.A., Cernin P.A., Leuchter A.F., Ganz P.A. (2013). Assessment of the feasibility of a rehabilitation intervention program for breast cancer survivors with cognitive complaints. Brain Imaging Behav..

[52] Green H.J., Tefay M., Mihuta M.E. (2018). Feasibility of small group cognitive rehabilitation in a clinical cancer setting. Psychooncology.

[53] Liang M.I., Erich B., Bailey C., Jo M.-Y., Walsh C.S., Asher A. (2019). Emerging from the Haze: A Pilot Study Evaluating Feasibility of a Psychoeducational Intervention to Improve Cancer-Related Cognitive Impairment in Gynecologic Cancer Survivors. J. Palliat. Care.

[54] Mariani M., George K. (2018). Neuropsychological and self-reporting outcomes following rehabilitation of cognitive dysfunction in survivors of breast cancer: A pilot study involving survivor-partner dyads. Breast J..

[55] Schuurs A., Green H.J. (2013). A feasibility study of group cognitive rehabilitation for cancer survivors: Enhancing cognitive function and quality of life. Psychooncology.

[56] Cherrier M., Anderson K., David D., Higano C., Gray H., Church A., Willis S. (2013). A randomized trial of cognitive rehabilitation in cancer survivors. Life Sci..

[57] Ercoli L.M., Petersen L., Hunter A.M., Castellon S.A., Kwan L., Kahn-Mills B.A., Embree L.M., Cernin P.A., Leuchter A.F., Ganz P.A. (2015). Cognitive rehabilitation group intervention for breast cancer survivors: Results of a randomized clinical trial. Psychooncology.

[58] Kesler S., Hosseini S.H., Heckler C., Janelsins M., Palesh O., Mustian K., Morrow G. (2013). Cognitive training for improving executive function in chemotherapy-treated breast cancer survivors. Clin. Breast Cancer.

[59] Mihuta M.E., Green H.J., Shum D.H.K. (2018). Web-based cognitive rehabilitation for survivors of adult cancer: A randomized controlled trial. Psychooncology.

[60] Meneses K., Benz R., Bail J.R., Vo J.B., Triebel K., Fazeli P., Frank J., Vance D.E. (2018). Speed of processing training in middle-aged and older breast cancer survivors (SOAR): Results of a randomized controlled pilot. Breast Cancer Res. Treat..

[61] Bray V.J., Dhillon H.M., Bell M.L., Kabourakis M., Fiero M.H., Yip D., Boyle F., Price M.A., Vardy J.L. (2017). Evaluation of a Web-Based Cognitive Rehabilitation Program in Cancer Survivors Reporting Cognitive Symptoms After Chemotherapy. J. Clin. Oncol..

[62] Goedendorp M.M., Knoop H., Gielissen M.F., Verhagen C.A., Bleijenberg G. (2014). The effects of cognitive behavioral therapy for post-cancer fatigue on perceived cognitive disabilities and neuropsychological test performance. J. Pain Symptom Manag..

[63] Ferguson R.J., Sigmon S.T., Pritchard A.J., LaBrie S.L., Goetze R.E., Fink C.M., Garrett A.M. (2016). A randomized trial of videoconference-delivered cognitive behavioral therapy for survivors of breast cancer with self-reported cognitive dysfunction. Cancer.

[64] Freeman L.W., White R., Ratcliff C.G., Sutton S., Stewart M., Palmer J.L., Link J., Cohen L. (2015). A randomized trial comparing live and telemedicine deliveries of an imagery-based behavioral intervention for breast cancer survivors: Reducing symptoms and barriers to care. Psychooncology.

[65] Johnston M.F., Hays R.D., Subramanian S.K., Elashoff R.M., Axe E.K., Li J.-J., Kim I., Vargas R.B., Lee J., Yang L. (2011). Patient education integrated with acupuncture for relief of cancer-related fatigue randomized controlled feasibility study. BMC Complement. Altern. Med..

[66] Zeng Y., Cheng A.S.K., Song T., Sheng X., Wang S., Xie J., Chan C.C.H. (2018). Effects of Acupuncture on Cancer-Related Cognitive Impairment in Chinese Gynecological Cancer Patients: A Pilot Cohort Study. Integr. Cancer Ther..

[67] Alvarez J., Meyer F.L., Granoff D.L., Lundy A. (2013). The effect of EEG biofeedback on reducing post-cancer cognitive impairment. Integr. Cancer Ther..

[68] Lawrence J.A., Griffin L., Balcueva E.P., Groteluschen D.L., Samuel T.A., Lesser G.J., Naughton M.J., Case L.D., Shaw E.G., Rapp S.R. (2016). A study of donepezil in female breast cancer survivors with self-reported cognitive dysfunction 1 to 5 years following adjuvant chemotherapy. J. Cancer Surviv..

[69] Bellens A., Roelant E., Sabbe B., Peeters M., van Dam P.A. (2020). A video-game based cognitive training for breast cancer survivors with cognitive impairment: A prospective randomized pilot trial. Breast.

[70] Smith T.M., Wang W. (2021). Comparison of a standard computer-assisted cognitive training program to a music enhanced program: A mixed methods study. Cancer Rep..

[71] Miladi N., Dossa R., Dogba M.J., Cléophat-Jolicoeur M.I.F., Gagnon B. (2019). Psychostimulants for cancer-related cognitive impairment in adult cancer survivors: A systematic review and meta-analysis. Support. Care Cancer.

[72] LaPointe L.L., Stierwalt J.A.G. (2018). Aphasia and Related Neurogenic Language Disorders.

[73] Karschnia P., Parsons M.W., Dietrich J. (2019). Pharmacologic management of cognitive impairment induced by cancer therapy. Lancet Oncol..

[74] Takemura N., Ho M.H., Cheung D.S.T., Lin C.C. (2022). Factors associated with perceived cognitive impairment in patients with advanced lung cancer: A cross-sectional analysis. Support. Care Cancer.

[75] Giannouli V. (2017). Predictive factors of depressive symptoms of elderly patients with cancer: A critical comment. Psychooncology.

